# Two 20-Residue-Long Peptides Derived from *Plasmodium vivax* Merozoite Surface Protein 10 EGF-Like Domains Are Involved in Binding to Human Reticulocytes

**DOI:** 10.3390/ijms22041609

**Published:** 2021-02-05

**Authors:** Laura Alejandra Ricaurte-Contreras, Andrea Lovera, Darwin Andrés Moreno-Pérez, Michel David Bohórquez, Carlos Fernando Suárez, Elizabeth Gutiérrez-Vásquez, Laura Cuy-Chaparro, Diego Garzón-Ospina, Manuel Alfonso Patarroyo

**Affiliations:** 1 Molecular Biology and Immunology Department, Fundación Instituto de Inmunología de Colombia (FIDIC), Carrera 50#26-20, Bogotá 111321, Colombia; lauraleja_9510@hotmail.com (L.A.R.-C.); yaloveras@unal.edu.co (A.L.); darandmorper@gmail.com (D.A.M.-P.); mdbohorqueza@unal.edu.co (M.D.B.); eliguva@hotmail.com (E.G.-V.); lauracuy@outlook.com (L.C.-C.); degarzon@gmail.com (D.G.-O.); 2 MSc Programme in Microbiology, Universidad Nacional de Colombia, Carrera 45#26-85, Bogotá 111321, Colombia; 3 Biomathematics Department, Fundación Instituto de Inmunología de Colombia (FIDIC), Carrera 50#26-20, Bogotá 111321, Colombia; cfsuarezm@gmail.com; 4 Health Sciences Division, Main Campus, Universidad Santo Tomás, Carrera 9#51-11, Bogotá 110231, Colombia; 5 Microbiology Department, Faculty of Medicine, Universidad Nacional de Colombia, Carrera 45#26-85, Bogotá 111321, Colombia

**Keywords:** *Plasmodium vivax*, surface molecule, *Pv*MSP10, protein-cell interaction, reticulocyte population

## Abstract

*Plasmodium* parasites’ invasion of their target cells is a complex, multi-step process involving many protein-protein interactions. Little is known about how complex the interaction with target cells is in *Plasmodium vivax* and few surface molecules related to reticulocytes’ adhesion have been described to date. Natural selection, functional and structural analysis were carried out on the previously described vaccine candidate *P. vivax* merozoite surface protein 10 (*Pv*MSP10) for evaluating its role during initial contact with target cells. It has been shown here that the recombinant carboxyl terminal region (r*Pv*MSP10-C) bound to adult human reticulocytes but not to normocytes, as validated by two different protein-cell interaction assays. Particularly interesting was the fact that two 20-residue-long regions (^388^DKEECRCRANYMPDDSVDYF^407^ and ^415^KDCSKENGNCDVNAECSIDK^434^) were able to inhibit r*Pv*MSP10-C binding to reticulocytes and rosette formation using enriched target cells. These peptides were derived from *Pv*MSP10 epidermal growth factor (EGF)-like domains (precisely, from a well-defined electrostatic zone) and consisted of regions having the potential of being B- or T-cell epitopes. These findings provide evidence, for the first time, about the fragments governing *Pv*MSP10 binding to its target cells, thus highlighting the importance of studying them for inclusion in a *P. vivax* antimalarial vaccine.

## 1. Introduction

Protein-protein interactions are one of the most important mechanisms used by microorganisms to recognise their target cells; they are mainly surface protein-mediated and can therefore be considered critical for developing control methods against parasites. Many studies to date have been related to surface molecules participating in early *Plasmodium falciparum* merozoite (Mrz) adhesion to red blood cells (RBC) [1]. However, studying proteins related to *Plasmodium vivax* Mrz adhesion or invasion has been difficult since it has not been possible to continuously propagate this parasite in vitro, given its preference for invading reticulocytes (cells occurring in low percentages in different sources) [2] for which high percentages cannot be obtained and also needing to be totally viable for supplementing cultures.

Despite this, several *P. vivax* Mrz surface proteins’ (merozoite surface protein 1 (MSP1), MSP1 paralogue (MSP1-P), reticulocyte binding surface protein (RBSA) and tryptophan-rich antigen (TRAgs)) binding to human RBC has been studied experimentally via different approaches. MSP1 is the most abundant glycosylphosphatidylinositol (GPI)-anchored *P. vivax* molecule which has high schizont/Mrz expression. This protein contains two epidermal growth factor (EGF)-like motifs which have been able to induce rosette formation on COS-7 cells [3,4]. It has been reported that MSP1 contains several 20-residue-long regions (i.e., peptides, one located in the EGF-like motif) having high specific reticulocyte interaction as evaluated by radio iodination tests [5]. MSP1-P shares the same genetic structural characteristics as MSP1. This molecule has a 19 kDa C-terminal region producing a large amount of rosettes on COS-7 cells [6]. RBSA is antigenic during natural infection and its reticulocyte binding activity has been confirmed by flow cytometry. This protein has a functional constraint region-derived peptide which has been able to inhibit RBSA attachment to target cells [7]. TRAg proteins (31 members) are encoded by the *Pv*-fam-a multigene family, predominantly transcribed in schizonts [8]. TRAg26.3, TRAg34, TRAg36 and TRAg36.6 have interacted with reticulocytes, as assessed by flow cytometry, cell-ELISA and rosetting assays [9].

Since Mrz surface is covered by a multitude of antigens, it is challenging to understand how initial adhesion to target cells occurs and how many molecules participate. Therefore, it is important to discover which other Mrz proteins or fragments derived from them play a role in reticulocyte binding, that is, the *P. vivax* merozoite surface protein 10 (*Pv*MSP10) vaccine candidate reported several years ago. This protein had a surface location pattern and high expression during schizont late stage [10]. The *pvmsp10* gene’s 3′-end region is conserved amongst species, possibly due to functional constraint [11,12]; this is consistent with two EGF-like motifs located just in the MSP10 carboxyl fragment. *Pv*MSP10 has been considered a biomarker as it is recognised by 90% of *P. vivax*-infected individuals’ sera [13] and has been suggested that it is a clinical protection-related antigen [14]. Furthermore, *Pv*MSP10 has triggered cytophilic antibody (Ab) production (i.e., IgG1 and IgG3) during natural infection which could have been related to host protection [15]. Interestingly, BALB/c mice immunised with recombinant *Pv*MSP10 expressed in a wheat germ cell-free system predominantly secreted Th1-type cytokines and elicited a robust immune response, thus supporting its potential as a vaccine candidate.

Despite this evidence, *Pv*MSP10 and/or minimal regions’ roles in cell binding have not been studied to date. This study has used natural selection along with functional and structural analysis to evaluate (for the first time) whether *Pv*MSP10 can interact with human reticulocytes and which minimum regions govern such function.

## 2. Results

### 2.1. The msp10 Gene’s Limited Diversity Is a Worldwide Characteristic

**Figure 1:**
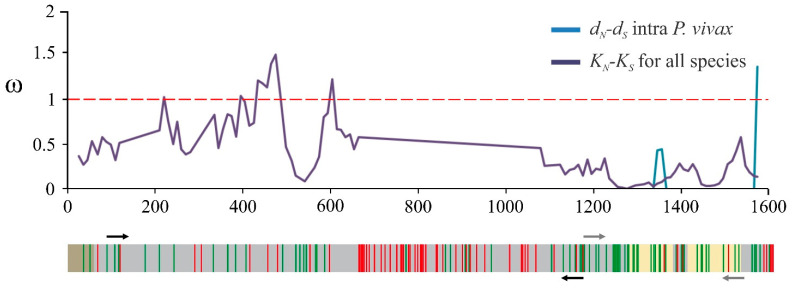
*msp10* sliding window showing ω rate (*y*-axis) and nucleotide position (*x*-axis); a gene model is shown. Codons under positive (red vertical lines) and negative (green vertical lines) selection are indicated, as well as *msp10-n* (black) and *msp10-c* (grey) primers (represented by arrows). Intra-*P. vivax*’ ω rate is represented by *d_N_*/*d_S_* whilst inter-*Plasmodium* species’ ω rate is denoted by *K_N_*/*K_S_*; ω values above 1 (red dotted line) are expected for positive selection (adaptative) whilst ω values lower than 1 are expected in negative (purifying) selection, the latter being due to functional constraint.

The high degree of *msp10* gene conservation observed in the Colombian population and laboratory strains [11,12] supported the notion that it encodes a protein playing an important role in parasite biology (i.e., target cell binding). Ninety-five whole gene sequences from different countries were thus analysed to confirm whether the *msp10* gene from worldwide populations behaved in the same way. The results showed that limited *msp10* diversity (π = 0.0012) is a worldwide feature (Supplementary data 1), seeming to be due to natural selection. Potential regions involved in parasite-host interaction were defined by inferring codons under negative (purifying) selection as not all protein sequences might be involved in parasite-host interaction and given that functionally important regions are conserved by purifying selection. More than 50% of the negatively selected codons were located in the *pvmsp10* gene’s 3′-end region (40 out of 72), which had a ω < 0.5 rate (Figure 1), this being expected behaviour for functionally important regions. Conversely, positively selected codons were mainly located in the central region (32/59) thereby supporting the idea that this region does not appear be involved in parasite-host interaction.

### 2.2. 3′-End Gene Region-Encoded PvMSP10-C Bound to Human Reticulocytes

The high degree of *msp10* gene conservation [11,12], the presence of codons under negative selection (mainly at the 3′-end gene region) and experimental evidence to date on *Pv*MSP10 [15] suggests that it plays an important role during initial parasite attachment to its target cells. *Pv*MSP10 (residues I^338^–Q^458^) and its fragments under (*Pv*MSP10-C (E^338^–E^458^)) or lacking (*Pv*MSP10-N (E^30^–E^333^)) functional constraint were thus obtained using an *Escherichia coli* system (Supplementary Figure S1a,b) to evaluate their reticulocyte binding function by flow cytometry. The purified molecules and enriched reticulocytes were useful for biological assays given that recombinant proteins did not cause lysis for erythrocytes incubated at 37 °C for one hour (Supplementary Figure S2a) and the cells were obtained in greater percentages (10–33%) (Supplementary Figure S2b) compared to that for the direct umbilical cord blood (UCB) samples previously used in functional assays (6–7%) [7,16].

**Figure 2:**
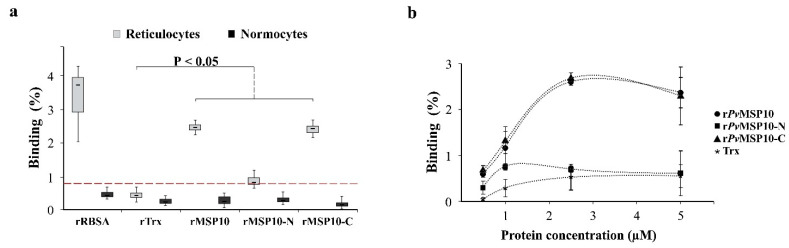
*Pv*MSP10 human adult reticulocyte binding activity. (**a**) Box diagram showing recombinant protein reticulocyte/normocyte binding percentages evaluated by flow cytometry and analysed with a Kruskal-Wallis test. Dotted line (red) indicates threshold positivity. (**b**) Saturation test indicating protein concentration (*x*-axis) and binding percentage (*y*-axis). SD for three independent assays are shown.

**Figure 3:**
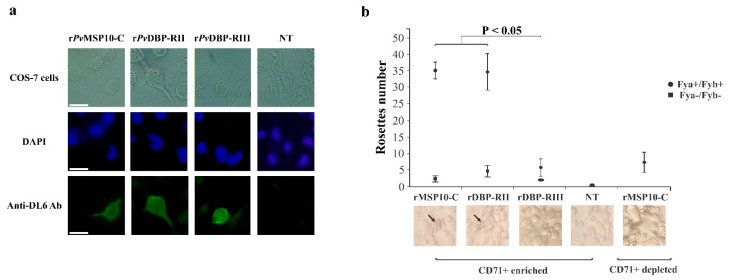
COS7 cells expressing r*P*vMSP10-C bound to human adult reticulocytes. (**a**) Immunofluorescence assay. r*Pv*MSP10-C, r*Pv*DBP-RII and r*Pv*DBP-RIII expression on COS-7 cell membrane verified by using anti-DL6 Ab. Cell nuclei were stained with 4′,6-diamidino-2-fenilindol (DAPI). Scale bar = 10 µm. (**b**) Erythrocyte binding assay showing the amount of rosettes formed on COS-7 cell surface expressing r*Pv*MSP10-C, r*Pv*DBP-RII and r*Pv*DBP-RIII. Assays were performed using a source of CD71+ enriched/depleted samples having positive (Fya+/Fyb+) or negative (Fya-/Fyb-) Duffy phenotype. A rosette observed using 40× magnification is indicated by an arrow in the bottom picture. Non-transfected (NT) cells were used as negative control. One factor ANOVA test was used for statistical analysis of three independent assays and Dunn-Bonferroni test for multiple comparisons among groups.

Binding analysis led to observing that r*Pv*MSP10 ($\bar{x}$ ± SD: 2.5 ± 0.12) and r*Pv*MSP10-C (2.4 ± 0.16) bound to human reticulocytes but not normocytes, having statistically significant differences compared to rTrx negative control (0.4 ± 0.12) (Figure 2a) according to post hoc comparison analysis; this suggested such molecules’ ability to interact with target cells. This interaction was similar to that for r*Pv*RBSA (3.3 ± 0.90), a protein previously validated as positive control [7]. It should be noted that such control’s binding percentage was lower than in previous work (6–9% compared to 3.3% found here) which could have been due to the amount of enriched reticulocytes in different maturation states in blood sources (high Heilmeyer class II and III group reticulocyte population in UCB compared to peripheral blood used in this study) [17]. Interestingly, r*Pv*MSP10 and r*Pv*MSP10-C reticulocyte binding percentages were similar (X^2^_(4)_ = 11.078, *p* = 0.855) and was concentration-dependent, reaching saturation at 2.5 µM (Figure 2b).

r*Pv*MSP10-C reticulocyte binding function was validated using a COS-7 eukaryotic system taking into account that such region contains two epidermal growth factor (EGF)-like domains (Accession ID: SSF57196) involved in parasite-host attachment in other Plasmodium species [18,19]. r*Pv*MSP10-C and controls were expressed correctly on COS-7 cell membrane (Figure 3a) and had 5% transfection efficiency. Rosette count for transfected COS-7 cells incubated with CD71+ enriched samples led to observing that r*Pv*MSP10-C was able to attach to Fya+/Fyb+ phenotype cells (34.8 ± 12.5), similar to r*Pv*DBP-RII (34.3 ± 9.8), having a statistically significant difference compared to negative control (r*Pv*DBP-RIII; 5.8 ± 2.7) (*p* = 0.007) (Figure 3b). By contrast, there were few rosettes for all proteins using Fya-/Fyb- phenotype cells and no statistical differences for their mean scores (r*Pv*MSP10-C: 2.3 ± 0.9; r*Pv*DBP-RII: 4.7 ± 1.7; r*Pv*DBP-RIII: 2.0 ± 0.2). Interestingly, a difference was found in rosettes’ mean averages for r*Pv*MSP10-C using normocytes as cell source (7.6 ± 11.5) compared to those for reticulocytes (34.8 ± 12.5), this being consistent with that observed by flow cytometry (Figure 1); this count was similar to that obtained for r*Pv*DBP-RIII binding to reticulocytes (F(1,4) = 0.06, *p* = 0.819). This data supports the idea that the *Pv*MSP10-C region is involved in binding to human reticulocytes.

### 2.3. PvMSP10 Binding Activity Was Governed by Two Small EGF-Like Domain-Derived Peptides

**Figure 4:**
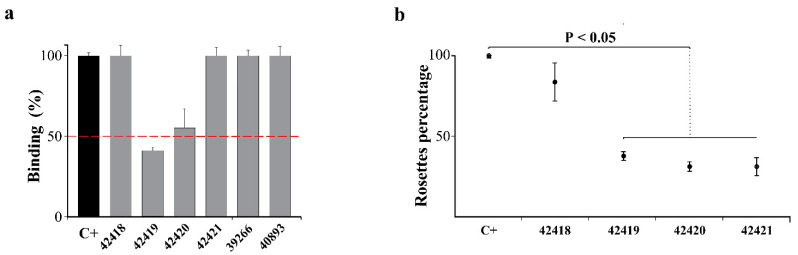
*Pv*MSP10-C human adult reticulocyte binding inhibition assay using EGF-like domain-derived peptides. Positive control (C+: r*Pv*MSP10) and peptides 42418, 42419, 42420 and 42421 are shown. Negative controls were 39266 *M. tuberculosis*- and 40893 *Pv*RBSA-derived peptides. All assays were done in triplicate and standard deviations are shown. Kruskal-Wallis (**a**) or Mann-Whitney (**b**) non-parametric tests were used for analysing statistical differences. The binding inhibition activity in panel a was considered as positive when each peptide reduced the r*Pv*MSP10-reticulocyte interaction by 50% (red dotted line).

An r*Pv*MSP10 human reticulocyte binding competition assay involved using four EGF-like domain-derived non-overlapping peptides (42418, 42419, 42420 and 42421) for determining the minimum region participating in protein-reticulocyte interaction. When the r*Pv*MSP10-reticulocyte interaction was competed by preincubating the cells with each peptide, there were only statistically significant differences for binding means’ data using peptides 42419 (1.2 ± 0.2) and 42420 (1.6 ± 0.3), compared to that for positive control (2.4 ± 0.15), according to post hoc test (H (7) = 22.55, *p* = 0.002) (Figure 4a). Control *M. tuberculosis* and *P. vivax* surface protein peptides could not inhibit r*Pv*MSP10 binding to cells. Interestingly, peptide 42419 (^388^DKEECRCRANYMPDDSVDYF^407^) reduced r*Pv*MSP10 binding to reticulocytes by 59% and 42420 (^415^KDCSKENGNCDVNAECSIDK^434^) by 45%.

Similar results were obtained in the rosette inhibition assay; the non-parametric test revealed differences amongst means for peptides 42419 (7.7 ± 0.5), 42420 (6.3 ± 0.47) and 42421 (6.3 ± 0.47) compared to that for positive control (20.3 ± 0.5) (U = 0.00, *p* < 0.05) (Figure 4b). Adding enriched reticulocytes pre-incubated with peptide 42419 to COS-7 cells expressing r*Pv*MSP10-C decreased rosette formation by 62% and 69% with peptide 42420 compared to control. Peptide 42421 results were not considered as inhibition activity, given that this mainly decreased COS-7 cell adherence to wells. Altogether, these findings thus supported the notion that protein-cell interaction between *Pv*MSP10 and human adult reticulocytes was governed by peptides 42419 and 42420.

### 2.4. Peptides 42419 and 42420 Are Promising Vaccine Candidates

**Figure 5:**
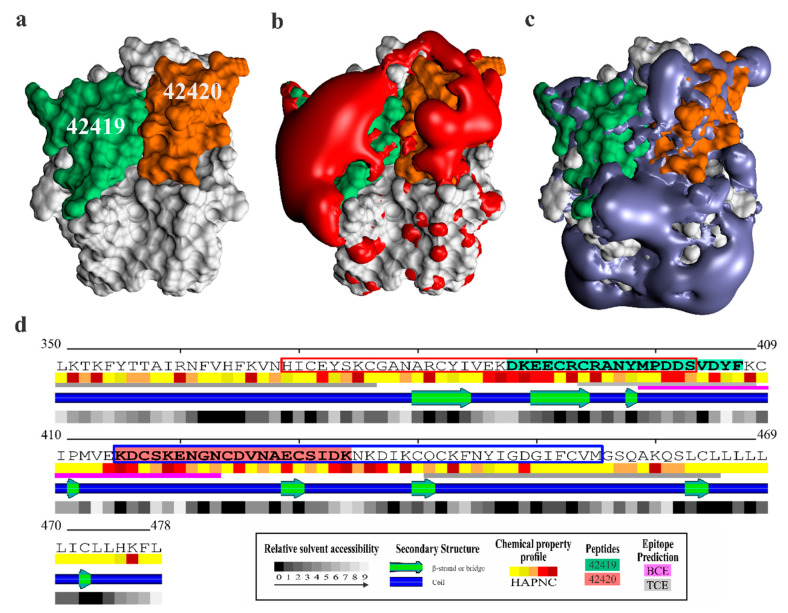
*Pv*MSP10-C_350–478_ structural and bioinformatics analysis. The figure shows peptide 42419 (green) and 42420 (orange) location (**a**), the domain’s electrostatic properties (potential isocontours −1 kT/e (red) (**b**) and +1 kT/e (blue) (**c**)) and secondary structure representation, including relative solvent accessibility (0: completely buried, 9: fully exposed), secondary structures, physicochemical amino acid profile (H: hydrophobic, A: amphipathic, P: polar, N or C: negative and positive charged), peptide location and regions containing B- or T-cell epitopes (**d**). EGF-like domains are framed within a red (EGF1, H^369^–S^403^) or blue (EGF2, K^415^–M^455^) box.

*Pv*MSP10-C (residues 350–378) was structurally modelled to analyse each peptide’s spatial location related to reticulocyte binding. The best *Pv*MSP10-C_350-478_ I-TASSER model showed similarity to structural analogues derived from *P. knowlesi* (RMSD 2.58 Å, 69.2% coverage and 28.9% identity), *P. cynomolgi* (RMSD 2.55 Å, 66.9% coverage and 28.7% identity) and *P. yoelii* (RMSD 2.80 Å, 66.9% coverage and 27.8% identity) MSP1-19 structures. A refinement protocol was used given that threading template identities were lower than 20%, enabling to obtain a structurally correct model meeting adequate quality criteria (Figure 5a and Supplementary Figure S3).

Peptides 42419 and 42420 were located on the same face and had a negative charge concentration pattern (Figure 5b,c). It is worth noting that both peptides had sectors whose composition was determined by charged aa having high relative solvent accessibility and regions containing short β-sheet organisation (Figure 5d). Particularly interesting was the observation that both peptides had sectors determined as being viable B-cell epitopes (Supplementary data 2). Only peptide 42419 consisted of potential T-cell epitopes for HLA-DRB1*03:02 (rank = 5.0%), HLA-A*02:01 (supertype A2) (rank = 0.35%), HLA-A*26:01 (supertype A26) (rank = 0.43%) and HLA-B*15:01 (supertype B62) (rank = 0.23%). These bioinformatics and structural findings highlighted these minimum regions’ importance for evaluating their usefulness as part of a subunit-based vaccine.

## 3. Discussion

Despite *P. vivax* proteins’ broad characterisation reported to date, the role of Mrz surface molecules in target cell binding remains relatively unknown [20]. This study was focused on evaluating *Pv*MSP10 human reticulocyte binding activity considering this and taking into account the importance of molecules involved in initial parasite-cell contact, the in silico findings reported for the *P. vivax msp10* gene [11,12] and evidence related to the r*Pv*MSP10-triggered immune response during natural and experimental infection [15].

It is well-known that proteins’ (or encoding genes) functional or structurally important regions tend to be conserved by purifying selection [21,22]. Low genetic polymorphism around the world on the *msp10* gene was thus confirmed, including all the sequences reported to date (Supplementary data 1). *pvmsp10* had less sequence diversity (π = 0.0012) than that of other Mrz surface proteins considered promising vaccine candidates, such as *pvmsp1* (π > 0.052) [23], *pvmsp3a* (π > 0.0349) [24], *pvmsp7* (-C, π = 0.0548; -E, π = 0.0390; -H, π = 0.0357; -I, π = 0.0430; -K, π = 0.0025) [25,26] or *pvrbsa* (π = 0.0080) [7]. Several codons, mainly derived from the 3′-end region (Figure 1), were under negative selection which would be an expected pattern for regions under functional constraint. The limited diversity and natural selection signatures identified in *msp10* led to inferring that the protein fragment encoded by the 3′-end region would be functionally important for the parasite and could be related to target cell attachment.

The reticulocyte binding activity of product encoding the *pvmsp10* gene 3′-end region (*Pv*MSP10-C) was evaluated in the light of these findings. The complete *pvmsp10* gene (*Pv*MSP10) and the 5′-end (*Pv*MSP10-N) region were also used to confirm the hypothesis that protein regions under constraint play functional roles. Although UCB has been the main reticulocyte source used in several *P. vivax* functional studies, peripheral blood was preferred as it represents the best approach to studying naturally-occurring parasite-host molecular interactions. Human adult-derived CD71+ reticulocytes were thus enriched by magnetic separation given *P. vivax* preference for this type of cell (Supplementary Figure S2b). According to the proposed hypothesis, it was found that *Pv*MSP10 cell binding was mediated by the protein fragment encoded by the gene’s 3′-end region (*Pv*MSP10-C) (Figure 2a). Interestingly, r*Pv*MSP10-C interacted only with human reticulocytes but not normocytes, whilst r*Pv*MSP10-N bound to target cells to a lesser amount. These results were consistent with those obtained using a COS-7 system, thereby confirming r*Pv*MSP10-C preference for human reticulocyte attachment (Figure 3b). It is likely that *Pv*MSP10 could interact with a specific receptor on their target cells (as confirmed by saturation test: Figure 2b) which decreases as cells mature [27]. It has been reported that the *Pv*MSP10 homologue protein in *P. falciparum* (*Pf*MSP10) binds to erythrocytes through several fragments (residues 188–507 including the EGF-like domain) whilst the N-terminal region (residues 29–188) participates in intramolecular interaction with *Pf*GAMA [28], indicating that the protein has functionally specialised regions. This supports the idea that *Pv*MSP10-C specialises in reticulocyte binding whilst *Pv*MSP10-N is probably involved in distracting the immune system, given its polymorphic nature and the high probability of consisting entirely of B-cell epitopes (Supplementary Figure S4a); future studies specifically examining *Pv*MSP10-N antigenicity could contribute to confirming this hypothesis.

A screening assay was carried out for determining the minimum regions involved in *Pv*MSP10-C binding to human reticulocytes as it has been reported that its *P. falciparum* homologue protein can attach itself to its target cells through 20-residue-long peptides [29]. Two peptides were able to inhibit r*Pv*MSP10-reticulocyte interaction (42419: D^388^-F^407^ and 42420: K^415^–K^434^), as validated by two different receptor-ligand interaction techniques (Figure 4). *Pv*MSP10 binding peptides were totally different to those reported for *Pf*MSP10 (K^421^–C^440^) (Supplementary Figure S4b) [29], supporting the notion that *P. vivax* uses different protein regions to interact with human reticulocytes. Each peptide was seen to have very differentiated electrostatic potential, having a marked negative charge concentration on protein surface, whilst the rest of the region had a positive charge (Figure 5b,c). It has been demonstrated that a protein’s electrostatic landscape plays a fundamental role in the molecular orientation and assembly required for receptor-ligand interaction [30,31], these being common characteristics in most vaccine candidates identified in *P. falciparum* [32]. It can thus be suggested that peptides 42419 and 42420 participate in common and vital *P. vivax* biology-related procedures (mainly parasite binding), given the evidence found here.

It should be stressed that peptides 42419 and 42420 were derived from EGF-like domains (Figure 5d) which had a cell binding function in other surface molecules [3]. It has been proposed that the MSP1 EGF-like domain’s load distribution enables host-parasite molecular interaction, the electrostatic landscape being important in such function [18]. This process is also promoted by zeta potential (net electrical charge) of RBC which increases during their maturation by changes in the composition of negatively charged sialic acid residues or lipids on the membrane [33,34]. Such differences in membrane charge could explain the parasites’ preferences for their target cells [35]. The above, added to the findings obtained here, thus highlights the fact that EGF-like domains are crucial elements in *P. vivax* merozoite interaction with target cells.

Evaluating the possibility of using such fragments as part of an antimalarial vaccine revealed that both peptides consisted of sites determined as B-cell epitopes. Particularly interesting was the fact that peptide 42419 had the dual characteristic of being a B- or T-cell epitope as it also had potential regions for binding to HLA class I and HLA class II molecules (Supplementary data 2). It has been found that Abs produced by *Aotus* primates after full r*Pv*MSP10 immunisation have failed to protect against blood-stage malaria [36]. This could be explained by fact that the r*Pv*MSP10 region being used was quite large and anti-*Pv*MSP10 Ab may not have been directed against the molecule’s functionally important regions. Studies evaluating 42419 and 42420 peptide binding specificity for human reticulocytes by radio iodination, their ability to inhibit parasite invasion in short-term in vitro cultures (or that of the Ab targeting them) and to induce a protective immune response are required to validate such hypothesis.

## 4. Materials and Methods

### 4.1. msp10 Genetic Diversity and Natural Selection

*msp10* gene polymorphism was assessed by using the data for 95 complete gene sequences from 10 countries available in PlasmoDB (Release 46, 19 November) [37]. The TranslatorX web server [38] was used for aligning the DNA sequences, using the MUSCLE method [39] for keeping the frame open. Maximum likelihood (internal fixed effects likelihood (IFEL) [40], fixed effects likelihood (FEL), single-likelihood ancestor counting (SLAC) [41], mixed effects model of evolution (MEME) [42]) and Bayesian methods (random effects likelihood (REL) [41] and fast, unconstrained Bayesian approximation (FUBAR) [43]), were used for assessing natural selection. *P. vivax* sequences with orthologous sequences from non-human parasites (*P. fragile* (GenBank accession number JOOM01000597.1, Cambridge, MA, USA), *P. knowlesi* (CWHR02000021.1, Thuwal, Saudi Arabia), *P. inui* (AMYR01000135.1, Cambridge, MA, USA), *P. cynomolgi* (BAEJ01001053.1, Osaka, Japan), *P. coatneyi* (CP016249.1, Athens, GA, USA) and *P. simiovale* (JQ286382.1, Tempe, Arizona, USA)) were thus used to infer codons under negative selection (this being a characteristic for predicting potential functional regions). The GARD genetic algorithm was used for detecting recombination [44] before running these tests [45,46]. DnaSP software [47] was used for inferring a sliding window for evolutionary rate (ω).

### 4.2. Plasmid Construction

Specific primers were manually designed using Gene Runner v3.05 software for polymerase chain reaction (PCR) amplification of regions encoding the *P. vivax* VCG-1 strain *Pv*MSP10 amino (*Pv*MSP10-N) (*msp10*-n-dir: 5′-TTCtggccaTATGGAATTGAATGGAACGGACG-3′; *msp10*-n-rev: 5′-TCCctcgagTTCCTTCAAGTCTATCCCC-3′) and carboxyl (*Pv*MSP10-C) fragments (*msp10*-c-dir: 5′-TTCtggccaTATGATCGATAACGCCGTGTAC-3′; *msp10*-c-rev: 5′-TCCctcgagCTGAGAACCCATGACGCA-3). *msp10*-n-dir and *msp10*-c-rev primers were used for obtaining the gene encoding the complete molecule without signal peptide and GPI anchor site sequences (referred to as *Pv*MSP10). PCR was carried out at 25 µL final volume containing 1x Phusion HSII High Fidelity Master mix, 0.3 µM of each primer and 10 ng *P. vivax* VCG-1 strain gDNA. Amplification consisted of an initial cycle at 98 °C for 30 s, followed by 35 cycles at 98 °C for 30 s, 56 °C for 20 s and 72 °C for 30 s and a final extension step at 72 °C for 2 min. The expected products (*pvmsp10* (1309 bp), *pvmsp10*-n (934 bp) and *pvmsp10*-c (385 bp)) were verified by agarose gel electrophoresis (prepared at 1.5% *w/v* and stained with SYBR safe) using Hyperladder-II and then purified using a Wizard genomic DNA purification kit (Promega, Madison, WI, USA).

Five µg of each product was digested with *MscI* and *AvaI* enzymes (underlined in each primer) at 37 °C for 4 h and then ligated into Pet32b+ vector with T4 ligase (enzymes and ligase were obtained from New England Biolabs, Ipswich, MA, USA); the NEBioCalculator was used for calculating molar ratios. Ligation involved incubation at 22 °C for 3 h, followed by 4 °C for 15 h and 70 °C for 10 min. Each product was transformed in *Escherichia coli* JM109 cells (Invitrogen, Carlsbad, CA, USA) by thermal shock and spread on ampicillin (100 μg/mL) containing agar plates. An UltraClean 6 Minute Mini Plasmid Prep kit (MOBIO, Carlsbad, CA, USA) was used for extracting plasmid from 3 colony forming units (CFU) grown on ampicillin selective agar plates which were bidirectionally sequenced with pet32b-Dir: 5′-CGGTGAAGTGGCGGCAA-3′ and pet32b-Rev: 5′-CCAAGGGGTTATGCTAGT-3′ primers by the Sanger method.

GeneScript biotech company services (Piscataway, NJ, USA) were used for designing the *Pv*MSP10-C (residues I^338^–Q^458^) and *Pv*DBP-RIII (residues A^598^–N^829^) region fragments with codon optimisation in humans for transfection assays. Codon adaptation index (0.96 and 0.93), codon usage bias adjustment (79% and 67%) and AG content adjustment (49.2% and 59.3%) parameters were improved for each gene; restriction enzymes and CIS-acting elements and repeat sequences were removed. Gene products were cloned into *PvuII*/*ApaI* restriction sites from pRE4 vector [48] in frame with the HSVgD signal sequence and transmembrane segment to express these fragments on COS-7 cell membrane. Four μg recombinant plasmid were dissolved in 20 µL sterilised water, heating the mixture at 50 °C for 15 min. Each product was then transformed in *E. coli* JM109 cells, spread on ampicillin selective agar plates and incubated at 37 °C for 16 h. After confirming the three CFU clones’ integrity by sequencing, plasmids were extracted using a DNA Plasmid HiPure purification kit (Invitrogen) and labelled as pRE4-r*Pv*MSP10 and pRE4-r*Pv*DBP-RIII. COS-7 cells transfected with plasmid pHVDR22 (which expressed r*Pv*DBP-RII) were used as positive control [49].

### 4.3. Obtaining and Purifying Recombinant Proteins

Sequencing-confirmed recombinant plasmids (*pvmsp10*-pet32b, *pvmsp10*-n-pet32b and *pvmsp10*-c-pet32b) were transformed in *E. coli* BL21-DE3 expression cells (Invitrogen), following the manufacturer’s recommendations. An inoculum was then grown in LB selective medium at 37 °C for 16 h with shaking at 260 rpm to express it in 500 mL volume for 4 h, adding 0.2 mM Isopropyl β-d-1-thiogalactopyranoside (IPTG). Bacteria were precipitated by spinning at 4000 rpm at 4 °C for 30 min and the pellet was separated into two parts for treating them with either buffer A (20 mM TRIS, 1 mM ethylenediaminetetraacetic acid (EDTA) and 500 mM NaCl) to extract soluble recombinant (r) proteins or B (6 M urea, 10 mM Tris-Cl and 100 mM NaH_2_PO_4_) to extract denatured ones. Sonication conditions for the soluble extraction of r*Pv*MSP10 (residues E^30^–Q^458^) and r*Pv*MSP10-N (residues E^30^–E^333^) consisted of using 40% amplitude for 0.2 s ON and 0.2 s OFF on ice. r*Pv*MSP10-C extraction consisted of incubation with buffer B supplemented with SIGMAFAST protease inhibitors (Sigma-Aldrich, St. Louis, MI, USA) and 100 µg/mL lysozyme at 4 °C for 16 h at 4 rpm in a tube rotator (Fisher Scientific, Waltham, MA, USA). Proteins extracted by both methods were separated by spinning lysed bacteria at 13,500 rpm for 1 h.

Immobilised metal ion affinity chromatography (IMAC) was used for purification using a prepacked HisTrap FF crude histidine-tagged protein purification column with the ÄKTA start preparative chromatography system (GE Healthcare, Chicago, IL, USA) for protein purification. The column was equilibrated with buffer C (20 mM NaHPO_4_, 500 mM NaCl and 10 mM imidazole, pH 7.4) before passing the sample (previously filtered through 0.2 µm membrane). Unbound proteins were eluted with 30 volumes of buffer C and weakly bound ones with 40 volumes of the same buffer supplemented with 0.1% Triton X114. r*Pv*MSP10-C elution involved four washings using buffer B with decreasing concentrations of urea (3 M, 1.5 M and without urea) containing 0.1 mM oxidised glutathione and reduced to 1 mM to promote molecule folding. Resin bound proteins were eluted using a 0–500 mM imidazole gradient with buffer D (20 mM NaHPO_4_, 500 mM NaCl, 500 mM imidazole, pH 7.4) in 2 mL fractions. Purification parameters were 0.5 MPa pressure and 0.5 mL/min flow rate. An aliquot of each fraction was treated in reducing conditions and then separated by weight on 12% SDS-PAGE, in duplicate, at 125 v for 2 h. Polyacrylamide gels were visualised by Coomassie staining whilst the others were transferred to a nitrocellulose membrane for 2 h at 10 v using Bio-Rad’s Trans-Blot SD semi-dry electrophoretic transfer cell. After 3 washes with phosphate buffered saline (PBS, pH 7.2) containing 0.05% Tween, the membrane was blocked for 1 h at RT with 5% (*w/v*) milk solution and then for 1 h with peroxidase-conjugated monoclonal anti-polyhistidine Ab at 1:4500 dilution (Sigma-Aldrich). The reaction was revealed using a peroxidase substrate kit (Vector Laboratories, Burlingame, CA, USA), following the manufacturer’s recommendations. Fractions in which a single band was observed by Coomassie staining and Western blot were dialysed in PBS and quantified using a BCA protein assay kit (Thermo Scientific, Rockford, IL, USA). r*Pv*RBSA [7] and thioredoxin (Trx) (incorporated in the pET32b + vector) were obtained in soluble conditions for use as controls in protein-cell interaction assays.

### 4.4. Ethics Committee and Reticulocyte Separation by Positive Selection

An adult human voluntarily signed an informed consent form after having received detailed information about the study’s purpose. A health worker then collected 5 mL of blood in a Vacutainer BD tube by venepuncture, following procedures approved by the Universidad del Rosario’s Research Ethics Committee (Code: DVO005 787-CV1096; Date: 19 June 2019). Duffy genotype was determined by standard blood banking method using Fya and Fyb Ab. The blood was homogenised 1:5 with phosphate-buffered saline (PBS) and passed through three cellulose columns (Invitrogen) to eliminate leukocytes and platelets. Then, 10^8^ cells were suspended in 800 µL PBS and incubated with 20 µL CD71 MicroBeads (Miltenyi Biotec, Bergisch Gladbach, Alemania) at 4 °C for 2 h at 4 rpm. Magnetically-labelled reticulocytes were gravity fed through a MS column coupled to a MiniMACS Separator. Unlabelled cells (CD71+ depleted fraction) were recovered by washing with PBS and the CD71+ cells (reticulocytes) were eluted by adding 500 µL PBS to the column previously removed from the magnetic separator. Washes were made thrice in each step with PBS, spinning at 300× *g* for 10 min. Cells were incubated with 200 µL Retic-Count for 20 min at room temperature (RT). 100,000 events were acquired in triplicate using 250 volts for forward scatter (FSC) and side scatter (SSC) and 440 volts for FITC fluorochrome on a FACSC Canto II cytometer (BD, Franklin Lakes, NJ, USA). Percentage reticulocyte enrichment was determined using the following gating strategy: doublet exclusion comparing FSC-H to FSC-A, cell selection according to granularity by plotting SSC-A against FSC-A and reticulocyte or normocyte population selection using the FITC signal (Emission at 530 as that for Retic-Count) against SSC-A (Supplementary Figure S5).

### 4.5. RBC Haemolysis Assay

10^8^ cells previously washed with PBS were incubated for 1 h at 37 °C with 5 μM of each recombinant protein and 100 μM of each synthetic peptide (see below) at 400 μL final volume using a tube rotator (Fisher Scientific). PBS containing 10% SDS was used as positive lysis control and 0% as negative control. Cells were precipitated by spinning at 13,000 rpm for 10 min at RT and 100 μL supernatant were transferred in triplicate to clear, flat-bottomed 96-well plates. Absorbance was measured at 540 nm using a plate reader. Negative control (PBS) background absorbance reading was subtracted from each reading and all data were normalised against average absorbance from maximum detergent treatment-induced haemolysis data. Haemolysis percentage for each individual test was then calculated, considering lysis from detergent control to be 100%.

### 4.6. Evaluating Protein-Cell Interaction by Flow Cytometry

r*Pv*MSP10, r*Pv*MSP10-N, r*Pv*MSP10-C, r*Pv*RBSA and Trx binding to human reticulocytes was evaluated in triplicate by flow cytometry. 10^6^ CD71+ enriched cells were incubated with different µM concentrations of each protein (0 to 5 µM) at 4 rpm for 16 h at 4 °C. Binding competition assays involved pre-incubating the cells with EGF-like domain-derived peptides located in *Pv*MSP10-C: peptide 1 (42418) in ^368^NHICEYSKCGANARCYIVEK^387^, peptide 2 (42419) in ^388^DKEECRCRANYMPDDSVDYF^407^, peptide 3 (42420) in ^415^KDCSKENGNCDVNAECSIDK^434^ and peptide 4 (42421) in ^435^NKDIKCQCKFNYIGDGIFCV^454^); they were then synthesised and purified (Supplementary Figure S6) as previously described [50]) in a 1:100 (protein:peptide) µM ratio at 4 °C for 1 h at 4 rpm. *Mycobacterium tuberculosis* (peptide 5 (39266): APSNETLVKTFSPGEQVTTY) and *Pv*RBSA (peptide 6 (40893): TASSESLAESNDAPSNSYESY) [7] -derived peptides were used as negative controls (all cysteine residues were replaced by serine to avoid peptide polymerisation). The samples were washed 3 times with PBS and then incubated with monoclonal Ab Anti-his tag APC conjugated mouse IgG1 clone AD1.1.10 diluted 1:50 (R&D systems, Minneapolis, MN, USA) and Retic-Count for 20 min in the dark. Protein-cell interaction was determined by analysing 100,000 events on a FACSCanto II cell analyser, using the aforementioned parameters. A gating strategy was used for selecting an RBC homogeneous population using FlowJoV10 software; reticulocyte binding percentage was analysed by plotting APC against FITC signals (Supplementary Figure S5). Average r*Pv*MSP10 binding to human adult reticulocyte percentage was considered 100%; all data obtained with individual peptides were compared to this positive control.

### 4.7. COS-7 Cell Maintenance and Transfection

COS-7 (American Type Culture Collection CRL-1651) cells were cultured in Dulbecco-Modified Eagle Medium (DMEM, Gibco, Co Dublin, Ireland) supplemented with 10% foetal calf serum (Gibco) at 37 °C with 5% CO_2_; 2 × 10^4^ COS-7 cells were then seeded and incubated in chamber slide sterile wells until reaching 40–60% confluence. Transfection involved using 300 ng *Pv*MSP10-C, *Pv*DBP-RIII or pHVDR22 plasmids with FuGENE HD reagent (Promega), following the manufacturer’s protocol. The cells were cultured in OptiMEM medium for 48 h in a humidified atmosphere containing 5% CO_2_ in an incubator at 37 °C. Immunofluorescence assays evaluated transfection efficiency. Briefly, transfected COS-7 cells were fixed with PBS containing 2% formaldehyde for 15 min at RT, blocked with 10% foetal calf serum (in PBS) for 1 h at RT and incubated with anti-DL6 Ab (Santa Cruz, Dallas, TX, USA) in 1:1000 dilution overnight at 4 °C and then with fluorescein-conjugated goat anti-mouse Ab for 1 h 30 min at RT in the dark. Each step involved three washes with PBS. Cell nuclei were stained with 0.1 µg/mL 4′,6-diamidino-2-phenylindole (DAPI) for 20 min; fluorescence was visualised on an Olympus BX51 microscope using an Olympus DP2 camera and Fiji software. Transfection efficiency percentage was calculated as total amount of fluorescent COS-7 cells × 100/total amount of COS-7 cells counted in 30 fields.

### 4.8. Erythrocyte Binding to COS-7 Transfected Cells Assay

100 µL reticulocytes (CD71+ enriched) or normocytes (CD71+ depleted) were added to wells containing transfected COS-7 cells and incubated for 2 h at 37 °C with 5% CO_2_. Plates were then washed three times with 2 mL PBS to remove non-adherent cells. The r*Pv*MSP10-C binding inhibition assay involved pre-incubating reticulocytes enriched with 10 µL peptides (1 mg/mL), in previously described conditions. The COS-7 cells with 50% of adhered cells on their surface (rosettes) were scored in 20 fields at 40× magnification under an inverted microscope. Each assay involved three independent experiments; non-transfected (NT) or pRE4-r*Pv*DBP-RIII transfected COS-7 cells were included as negative controls. Binding inhibition percentage was estimated considering the average percentage of r*Pv*MSP10 binding as 100%; all data obtained with individual peptides were normalised against this positive control.

### 4.9. Statistical Analysis

A Shapiro-Wilk test was used for analysing data normality from three experiments and a Levene test for variance. One factor ANOVA or Kruskal-Wallis or Mann-Whitney non-parametric methods were used for comparing differences between each group’s means (m) and Dunn-Bonferroni post hoc test for comparing groups. IBM SPSS software significance level was 0.05.

### 4.10. rPvMSP10-C_350-478_ 3D Structural Modelling and Bioinformatics Analysis

The iterative threading assembly refinement (I-TASSER) method [51] was used for predicting *rPv*MSP10-C_350-478_ 3D structure, which was then refined by 0.25 ns minimisation, followed by 2.5 ns molecular dynamics and NAMD 2.12 0.25 ns minimisation [52] using CHARMM36 protein force field [53] and an isotherm/isobar assembly (P = 1 atm, T = 300 K) using a TIP3 solvation model [54] and periodic boundary conditions. PROPKA [55] was used for estimating pKa per residue. MOLPROBITY and QMEAN4 [56,57] were used for assessing structure quality. PDB2PQR [58] was used for calculating charge and radius per atom; electrostatic potential was calculated from these results using adaptive Poisson-Boltzmann solver (APBS) (VMD APBS Plugin, version 1.3.1) [59]. The resulting r*Pv*MSP10-C_350-478_ model was visualised by VMD 1.9.3 [60]. Analysed peptides were located in the structure and POLYVIEW [61] was used for calculating relative solvent accessibility, aa physicochemical profile and secondary structure annotation. InterPro 78.0 [62] was used for domain prediction. BepiPred-2.0 (0.6 epitope threshold) [63] was used for calculating potential B-cell epitopes (BCE) and NetMHCpan 4.0 (class I, using HLA supertype representative, ≤0.5% rank threshold for strong binders and ≤2% for weak binders) [64] and NetMHCIIpan 3.2 (class II with most frequently occurring HLA-II alleles, ≤2% rank threshold for strong binders and ≤10%for weak binders) [65] for calculating T-cell epitopes (TCE).

## 5. Conclusions

This is the first study to show that the *pvmsp10* low polymorphism region encodes two functionally important regions related to parasite binding to its target cells: peptides ^388^DKEECRCRANYMPDDSVDYF^407^ and ^415^KDCSKENGNCDVNAECSIDK^434^. These regions had codons under negative selection and were derived from the EGF-like domains whose role in *Plasmodium* invasion of erythrocytes has been experimentally determined. It was found that these peptides were located in a conserved electrostatic area and had regions which could be B- or T-cell epitopes. Altogether, these findings highlighted such peptides’ possible use as subunit-based vaccine components. Further studies are required and should be focused on evaluating these minimum regions’ usefulness in a vaccine targeting *P. vivax* malaria blood stage.

## Data Availability

The data presented in this study are available within the article and in the Supplementary Material.

## Supplementary Materials

The following are available online at https://www.mdpi.com/1422-0067/22/4/1609/s1.

## Abbreviations

*P. falciparum*	*Plasmodium falciparum*
*P. vivax*	*Plasmodium vivax*
RBC	red blood cell
Mrz	merozoite
MSP1	merozoite surface protein 1
MSP1-P	MSP1 paralogue
RBSA	reticulocyte binding surface protein
TRAgs	tryptophan-rich antigen
GPI	glycosylphosphatidylinositol
EGF	epidermal growth factor
*Pv*MSP10	*P. vivax* merozoite surface protein 10
*Pv*MSP10-N	*P. vivax* merozoite surface protein 10 amino terminal region
*Pv*MSP10-C	*P. vivax* merozoite surface protein 10 carboxyl terminal region
*E. coli*	*Escherichia coli*
*Pf*MSP10	*Plasmodium falciparum* merozoite surface protein 10
CFU	colony forming unit
r	recombinant
IMAC	immobilised metal ion affinity chromatography
PBS	phosphate buffered saline
DAPI	4′,6-diamidino-2-phenylindole
I-TASSER	iterative threading assembly refinement
BCE	B-cell epitope
TCE	T-cell epitope
aa	amino acid
