# Supplementary material for: Two 20-Residue-Long Peptides Derived from Plasmodium vivax Merozoite Surface Protein 10 EGF-Like Domains Are Involved in Binding to Human Reticulocytes
Source: Int J Mol Sci. 2021 Feb 5;22(4):1609. doi: 10.3390/ijms22041609 (PMC7915351; doi:10.3390/ijms22041609)
Supplement: Supplementary file 1 [file ijms-22-01609-s001.pdf]

Supplementary files

1 Supplementary data

Supplementary data 1: *P. vivax msp10* gene genetic diversity

Isolate	*n*	*Sites*	*Ss*	*S*	*Ps*	*H*	θw *(SD)*	π *(SD)*
Worldwide	*95*	*1,437*	*18*	*8*	*10*	*14*	*0.0024 (0.0006)*	*0.0012 (0.0002)*
Colombian	*24*	*1,437*	*3*	*3*	*0*	*2*	*0.0006 (0.0003)*	*0.0002 (0.0006)*
Mexico	*14*	*1,437*	*0*	*0*	*0*	*1*	*0*	*0*
Peru	*19*	*1,437*	*0*	*0*	*0*	*1*	*0*	*0*
Brazil	*4*	*1,437*	*0*	*0*	*0*	*1*	*0*	*0*
China	*5*	*1,437*	*4*	*3*	*1*	*4*	*0.0013 (0.0007)*	*0.0012 (0.0004)*
Thailand	*11*	*1,437*	*4*	*0*	*4*	*3*	*0.0009 (0.0005)*	*0.0014 (0.0002)*
India	*2*	*1,437*	*0*	*0*	*0*	*1*	*0*	*0*
Cambodia	*3*	*1,437*	*3*	*3*	*0*	*2*	*0.0014 (0.0008)*	*0.0014 (0.0007)*
Papua New Guinea	*5*	*1,437*	*8*	*4*	*4*	*5*	*0.0027 (0.0009)*	*0.0028 (0.0008)*
Madagascar	*3*	*1,437*	*1*	*1*	*0*	*2*	*0.0005 (0.0005)*	*0.0005 (0.0005)*

Values for countries having two or more sequences available are shown. n: amount of sequences; Ss: amount of segregating sites; S: amount of singleton sites; Ps: amount of parsimonious sites; H: amount of haplotypes; θw: Watterson estimator; π: nucleotide diversity; SD: standard deviation.

Supplementary data 2: *Pv*MSP10$_{350\text{-}478}$ B- and T-cell epitope prediction

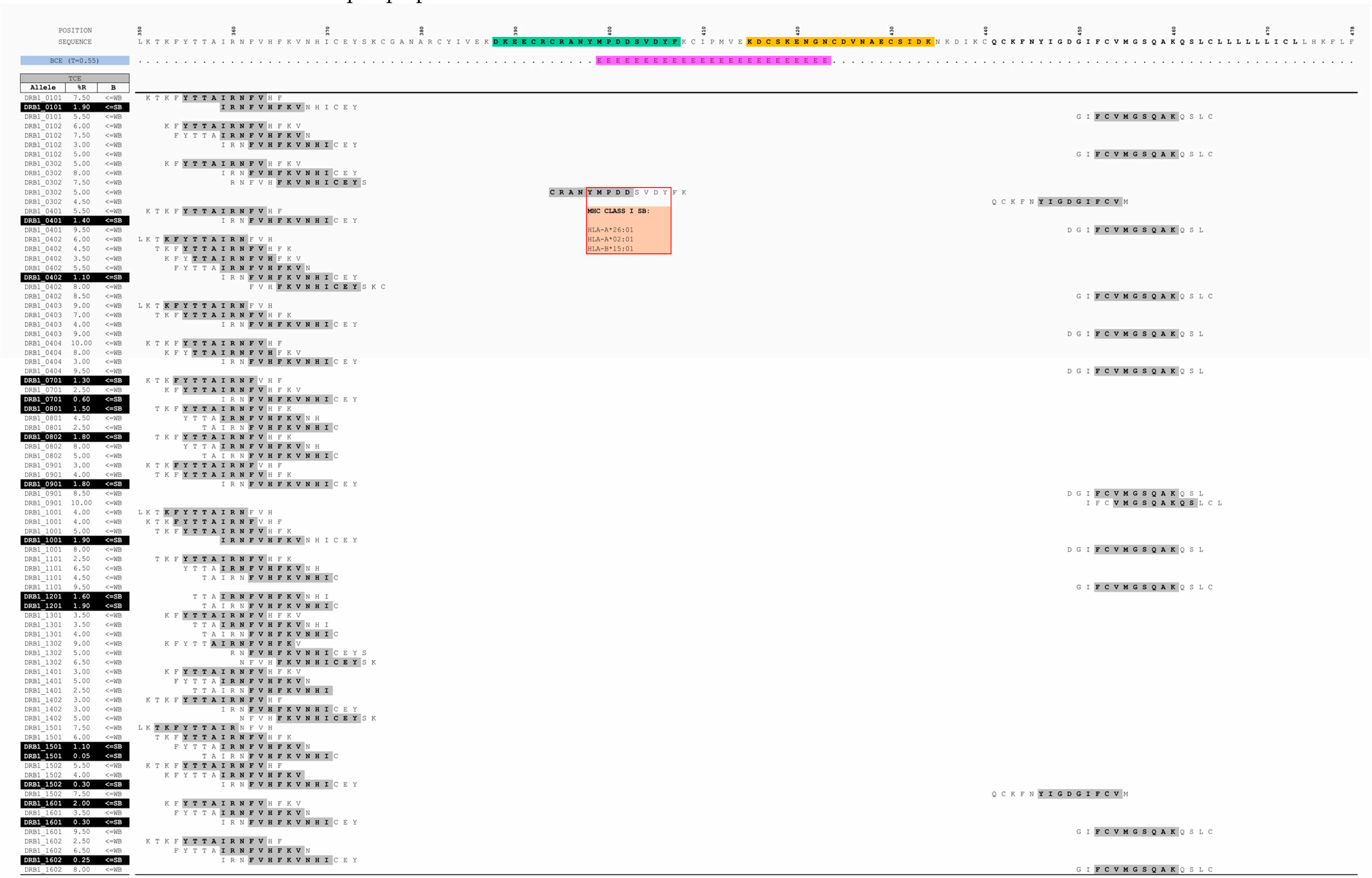

Peptide 42419 residues shaded in green and 42420 in orange, B-cell epitopes in magenta and T-cell epitopes in grey (HLA-DR class II cores). The red box shows the TCE for HLA-A and HLA-B (class I) molecules. WB: weak binder. SB: strong binder.

2 Supplementary Figures

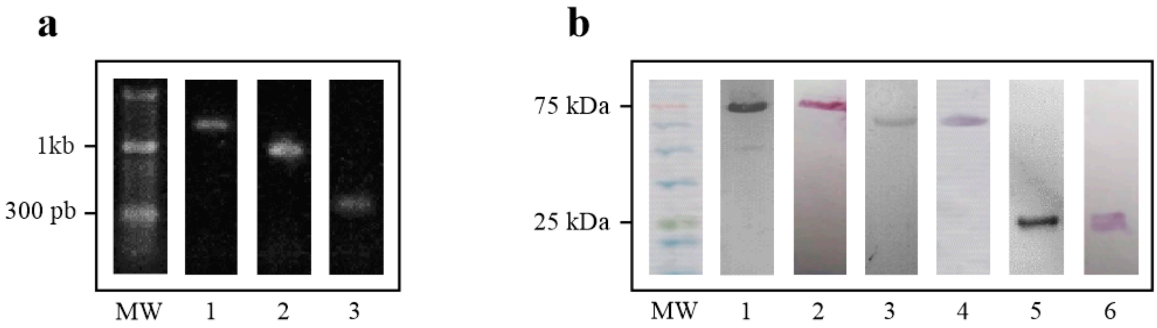

Supplementary Figure 1: *Pv*MSP10 fragment expression and purification. (a) pvmsp10 fragment amplification by PCR. Lanes 1 to 3 show the complete pvmsp10 gene and 5′-end and 3′-end regions. (b) PvMSP10, PvMSP10-N and PvMSP10-C purification analysed by Coomassie blue staining (odd lines) or Western blot (even lines). MW: molecular weight marker.

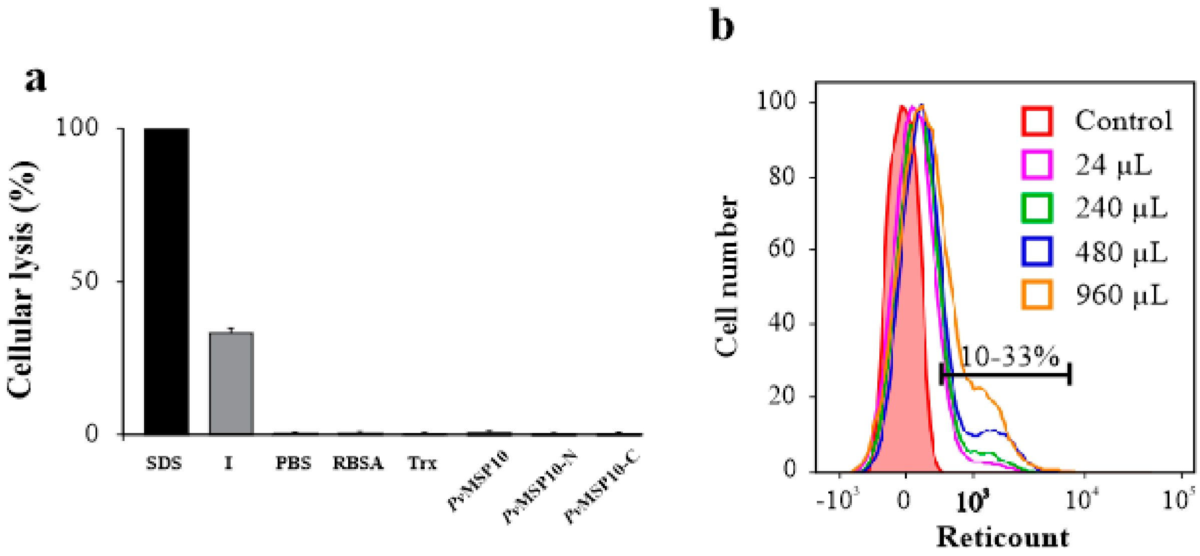

Supplementary Figure 2: Cellular haemolysis test and reticulocyte enrichment. (a) Erythrocyte lysis test in triplicate with 5µM of each recombinant protein (x axis). Assays carried out with 10% SDS (positive control) and buffer D containing imidazole (I) are shown. (b) Flow cytometry analysed reticulocyte enrichment percentage. The volume of blood used from adult humans and cell purification percentages are indicated. The result is representative of three independent experiments.

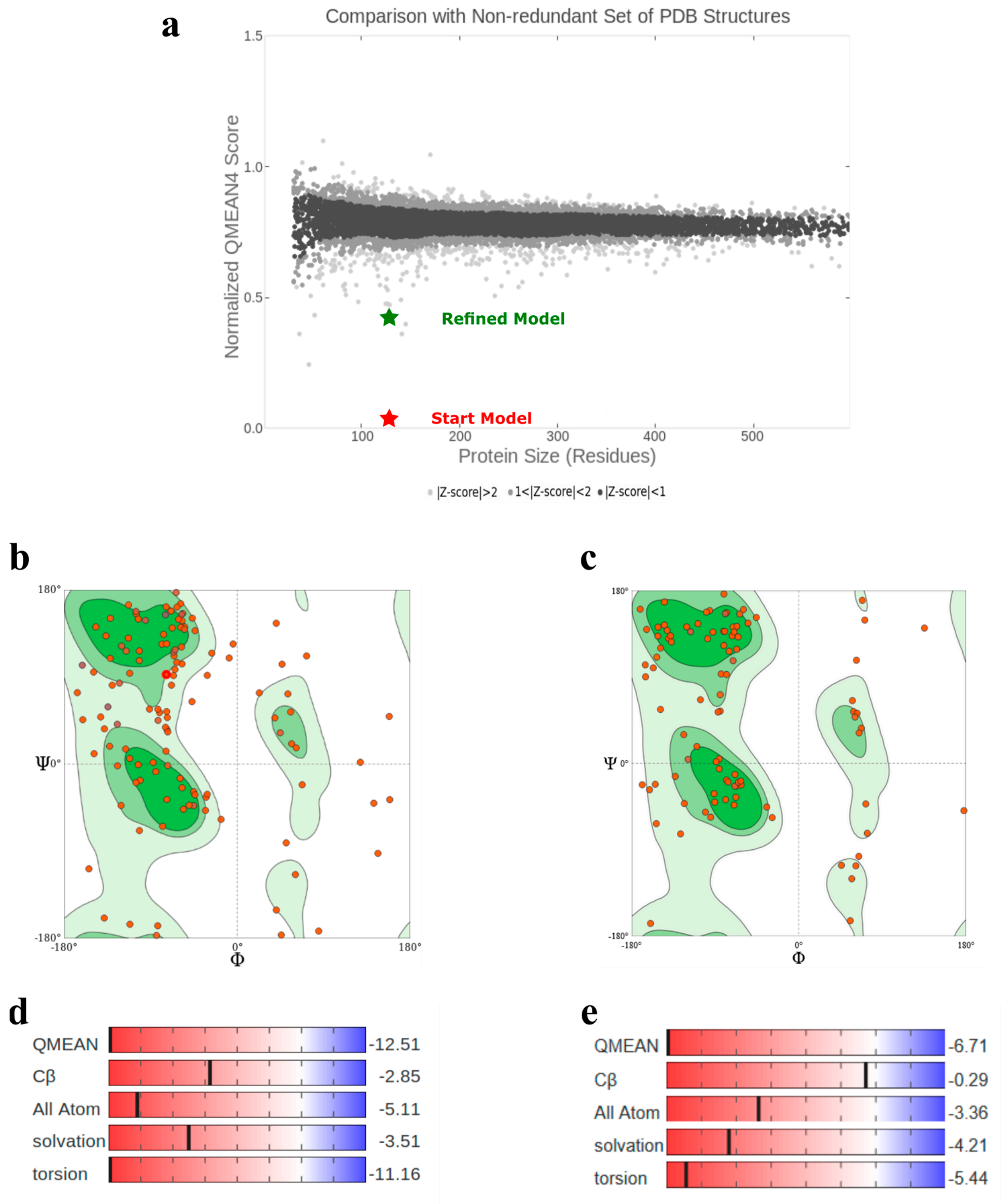

f

MolProbity Score 4.1
Clash Score **42.49**
Ramachandran Favoured 51.56%
Ramachandran Outliers 17.19%
Rotamer Outliers 22.69%
C-Beta Deviations 31
Bad Bonds 6 / 1065
Bad Angles 68 / 1430
Cis Prolines 1 / 2
Twisted Non-Proline 30 / 127

g

MolProbity Score 1.97
Clash Score **0.0**
Ramachandran Favoured 68.0%
Ramachandran Outliers 3.9%
Rotamer Outliers 6.3%
C-Beta Deviations 4
Bad Bond 2/1065
Bad Angles 20/1430
Cis Prolines 1 / 2
Twisted Non-Proline 1/129

Supplementary Figure 3: *Pv*MSP10$_{350\text{-}478}$ structure refinement. (a) Normalised QMEAN4 comparing the studied models to reported crystallographic structures. Note how the refinement brings the start homology model (red) closer to acceptable structural parameters (green). Ramachandran plot (b and c), QMEAN4 (d and e) and MOLPROBITY (f and g) quality parameters are shown for the initial and refined model, respectively.

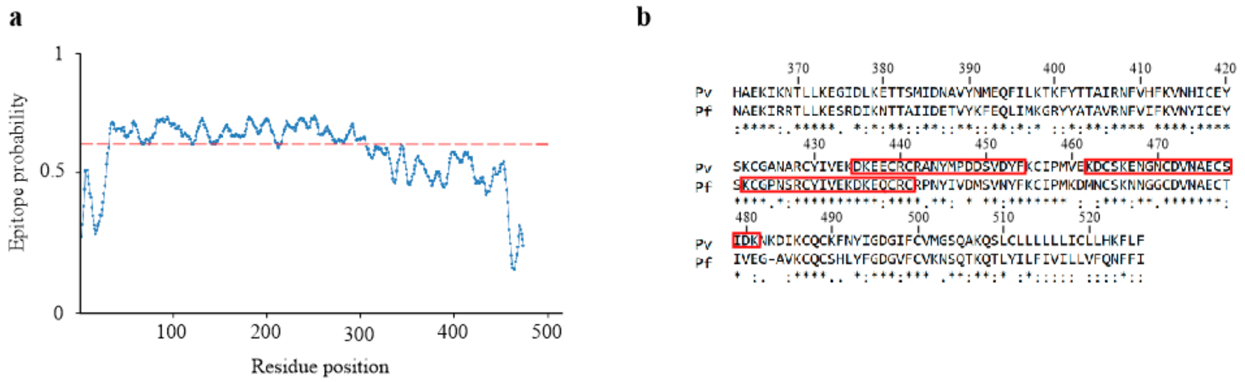

Supplementary Figure 4: *Pv*MSP10 B-cell epitope prediction and comparison with the *Pf*MSP10 aa sequence. (a) The BepiPred-0.2 predictor (available online) was used for analysing the *Pv*MSP10 aa sequence. The x-axis indicates residue number and y-axis the probability of being a B-cell epitope. Accordingly, *Pv*MSP10-N contained residues having higher scores than *Pv*MSP10-C, thus indicating that such region could be more antigenic. Positivity threshold was considered to be above 0.6 (dotted red line). (b) *P. vivax* and *P. falciparum* species MSP10$_{19}$ (containing EGF-like domains) alignment. Asterisks indicate identical residues, colons strongly similar ones and single dots weakly similar ones. Residue position is shown on top of the alignment. The red box shows target cell binding activity to fragments in *P. falciparum* (*Pf*) and *P. vivax* (*Pv*).

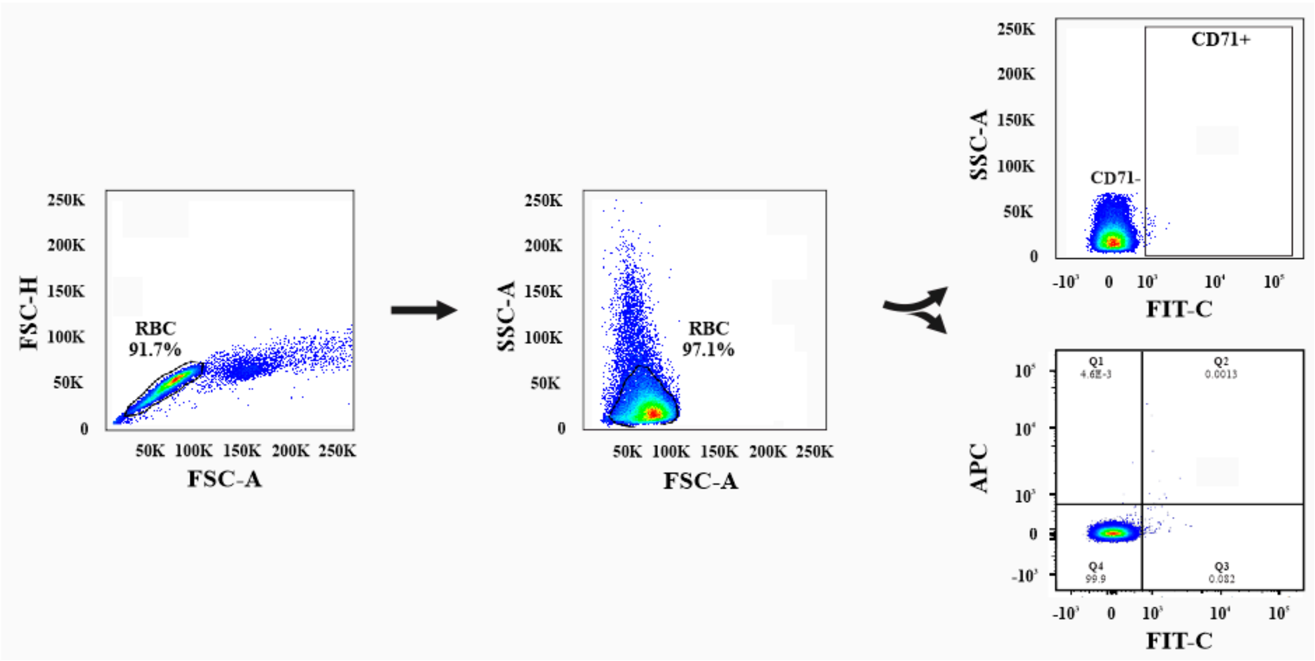

Supplementary Figure 5: Gating strategy for selecting reticulocyte or normocyte cell populations. Control cell population selection starting with doublet exclusion, comparing FSC-H to FSC-A, cell selection by cell granularity, plotting SSC-A against FSC-A and reticulocyte (CD71+)/normocyte (CD71-) population selection comparing the FIT-C signal to SSC-A. Percentage protein binding to normocytes (Q1) or reticulocytes (Q2) was analysed by plotting the APC against FIT-C signals.

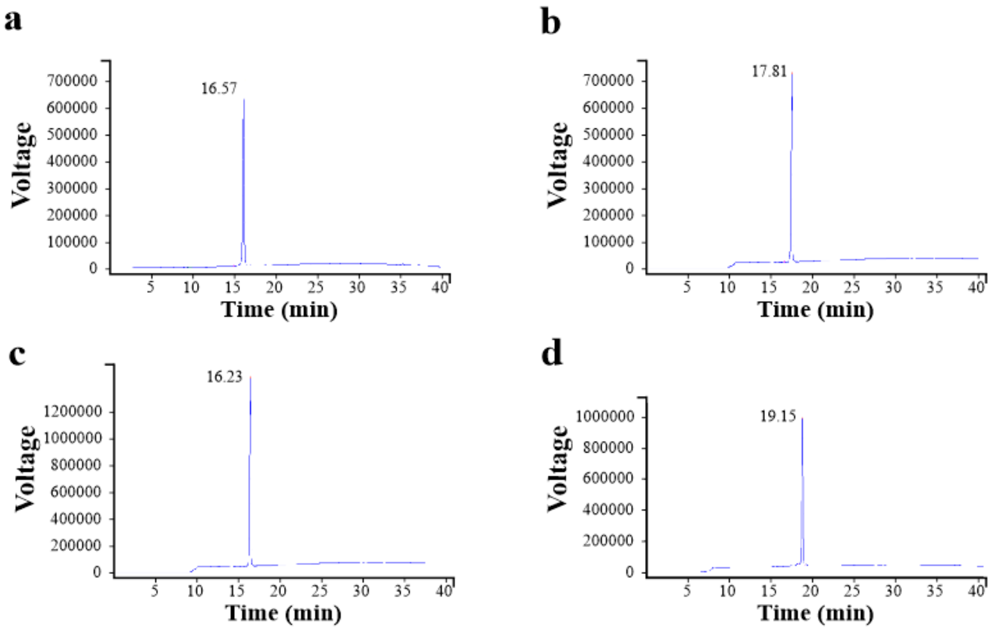

Supplementary Figure 6: Purified peptide chromatograms. Elution time (x-axis) and voltage (y-axis) are shown for purified peptides 42418 (a), 42419 (b), 42420 (c) and 42421 (d).
